# Diagnostic value of prothrombin induced by the absence of vitamin K or antagonist-II (PIVKA-II) for early stage HBV related hepatocellular carcinoma

**DOI:** 10.1186/s13027-017-0153-6

**Published:** 2017-08-23

**Authors:** Xiumei Wang, Weiwei Zhang, Youde Liu, Wenjing Gong, Ping Sun, Xiangshuo Kong, Miaomiao Yang, Zhihua Wang

**Affiliations:** 1 Department of Oncology, Yantai Yuhuangding Hospital, Yantai, Shandong 264000 People’s Republic of China; 2 Department of Gastroenterology, Yantai Yuhuangding Hospital, Yantai, Shandong 264000 People’s Republic of China; 3 Department of Hepatology, Infectious Disease Hospital of Yantai City, Yantai, Shandong 264001 People’s Republic of China

**Keywords:** Hepatocellular carcinoma, Hepatitis B virus, PIVKA-II, Early diagnosis

## Abstract

**Background:**

To evaluate the diagnostic efficacy of prothrombin induced by the absence of vitamin K or antagonist-II (PIVKA-II) for early stage hepatitis virus B (HBV) related hepatocellular carcinoma (HCC).

**Methods:**

Serums levels of PIVKA-II and a-Fetoprotein (AFP) was detected and compared in 113 patients with clinical confirmed Barcelona Clinic Liver Cancer (BCLC) stage 0-A HBV-related HCC and 161 chronic hepatitis B (CHB) patients. Diagnostic efficiencies as well as cut-off values of PIVKA-II, AFP and combination of the two markers were calculated using receiver operator curve (ROC) analysis.

**Results:**

The mean level of PIVKA-II among HCC patients were 79.64 ± 149.88, significantly higher than control group (*P* < 0.001). ROC results showed that among those AFP-negative HCC patients, the area under ROC curve (AUROC) of PIVKA-II was 0.73 (95%CI 0.640–0.815, *P* < 0.001). Among HCC patients diagnosed with small HCC (tumor size ≤2 cm), the AUROC of PIVKA- II was 0.692 (95%CI 0.597–0.788, *P* < 0.001). To evaluate the diagnostic value of PIVKA-II in HCC patient, all CHB cases were pooled together as control for analysis. The AUROC of PIVKA-II was 0.756 (95%CI 0.698–0.814, *P* < 0.001), and the optimal cutoff value of PIVKA-II was 32.09 mAU/ml with sensitivity of 52.21% and specificity of 81.49%. When serum levels of PIVKA-II and AFP were combined to obtain a new marker for HCC diagnosis, PIVKA-II + AFP further increased diagnostic efficiency, with AUROC of 0.868 (95%CI 0.822–0.913), higher than that of AFP (*P* < 0.01) or PIVKA-II (*P* < 0.001) alone. In addition, we found that HCC patients in poorly differentiated- undifferentiated group and in microvascular invasion group had higher levels of PIVKA-II. Multivariate analysis showed that high serum PIVKA-II level (OR = 1.003, 95%CI 1.001–1.007, *P* = 0.047) was an independent risk factor for microvascular invasion in HCC patients.

**Conclusion:**

Serum PIVKA-II level is a potential marker for early diagnosis of HCC and microvascular invasion. The use of PIVKA-II may improve assessment of tumor prognosis and guide development of therapeutic strategy.

## Background

Hepatocellular carcinoma (HCC) ranks fifth in worldwide prevalence of malignant tumors and second in frequency of cancer related death. The general prognosis of patients with HCC is poor, which might be due to the late diagnosis of HCC. For the patients diagnosed at early stage of HCC, the 5-year survival rate is >70% [1]. Diagnosis of early stage HCC is heavily dependent on radiological imaging technique, which does not provide sufficient sensitivity. Biological markers in serum may facilitate the early diagnosis of HCC, thus improving survival rate. Currently, serum alpha-fetoprotein (AFP) level is the most commonly used biomarker for HCC. However, the sensitivity and specificity of diagnosis at early stage of HCC are still far from ideal [2]. There have been efforts in identification of new biomarkers for the early diagnosis of HCC. Various factors such as des-γ-carboxyprothrombin, AFP-L3, glypican-3, osteopontin, Golgi protein-73 and a number of microRNAs have been suggested to be potential markers [1, 3].

Etiologies of HCC must be taken into consideration when choosing diagnostic biomarkers of HCC, since there are various pathological causes of HCC. It has been reported that chronic infection of hepatitis virus accounts for the majority of HCC development. However, the virus infection status varies significantly in different countries. Hepatitis B virus (HBV) infection has been shown to be mostly common in East Asia including China and HBV infection accounts for the major cause of HCC [4–6]. Approximately 240 million people worldwide are chronically infected with HBV and these people are at an increased risk of developing end-stage liver diseases, including cirrhosis and HCC [7–9]. Differential diagnosis of HBV related HCC patients and chronic HBV infected non-HCC patients is needed clinically. Therefore, the biomarkers have to reflect different pathological mechanisms of HBV infection [10].

Levels of prothrombin induced by vitamin K absence-II (PIVKA-II) have been identified to be elevated in HCC patients. Multiple studies have shown that PIVKA-II has high sensitivity and specificity for diagnosis of HCC. Although PIVKA-II is mainly used as a diagnostic marker in Asia, studies in other populations, including Middle Eastern and European subjects, have also demonstrated its diagnostic value for HCC [11–13]. There has also been report that combination of PIVKA-II with other biomarkers may further increase the sensitivity and specificity for early diagnosis of HCC [14]. The PIVKA-II level has also recently drawn interest as a prognostic marker for HCC patients [15]. Despite of the relatively conclusive recognition of PIVKA-II as HCC biomarker, the specific diagnostic performance on HBV infection related HCC is still uncertain. For example, although it has been suggested that hepatitis virus infection had no apparent influence, commonly existed hyperbilirubinemia in HBV related hepatitis and liver cirrhosis may cause dysfunction of vitamin K absorption, resulting in different PIVKA-II level, which is different from hepatitis C virus infection.

The current study aims to evaluate the performance of PIVKA-II, as well as PIVKA-II combined with AFP, in the diagnosis of HBV-related early stage of HCC.

## Methods

### Study settings and patients

Enrolled subjected included patients administrated from January 2014 to March 2015 in Yantai Yu Huang top Hospital and Infectious Disease Hospital of Yantai City. Patients were allocated into two different categories: HBV-related HCC patients (HCC group) and chronic HBV infected non-HCC patients (CHB group). According to Barcelona Clinic Liver Cancer stage (BCLC), 38 patients in HCC group were at BCLC stage 0 and 75 patients were at BCLC stage A. BCLC stage 0 is defined as a single lesion ≤ 2 cm, and BCLC stage A is defined as single lesion between 2 and 5 cm or ≤3 lesions with each lesion ≤3 cm. In CHB group, there are two kinds of people: patients with cirrhosis and patients without cirrhosis. A Total of 113 subjects were included in HCC group and 161 subjects were included in CHB group. The baseline characteristics of the two groups of patients were listed in Table 1.

**Table 1:** Patient information and baseline characteristics

	HCC	CHB	*P* value
Sample size	113	161	
Gender			0.435
Male	104	152	
Female	9	9	
Cirrhosis			0.199
Yes	80	102	
No	33	59	
Age, years	48 (28–70)	50 (21–75)	0.237
Lab			
ALT	74.02 ± 38.83	77.89 ± 84.87	0.650
AST	78.97 ± 46.59	69.64 ± 42.54	0.087
ALB	38.04 ± 10.97	40.36 ± 5.64	0.041
TBIL	27.13 ± 11.65	14.03 ± 7.57	<0.001
PLT	154.94 ± 69.67	144.96 ± 47.15	0.158

The diagnosis of liver cancer was made in accordance with the standards of diagnosis and treatment of primary liver cancer (2011 Edition) issued by the Ministry of public health of the People’s Republic of China [16]. The diagnosis of Chronic hepatitis B infection and cirrhosis was in accordance with the revised guidelines for the prevention and treatment of chronic hepatitis B infection (2015 Edition) [17] issued by Chinese Society of Hepatology. The study was approved by ethical committees of both participating hospitals and all study subjects signed informed consent.

### Measurement of PIVKA- II and AFP levels

Fasting venous blood (2 ml) samples were collected from HCC and CHB patients right before surgeries or other treatments. Serum samples were then separated and kept at −70 °C until laboratory tests.

PIVKA-II level was detected with a PIVKA-II reagent kit (FUJIREBIO Inc., Japan) on a LUMI-PULSE g1200 automatic immune analyzer according to manufacturer’s manual (FUJIREBIO Inc. Japan). AFP level was detected with an AFP reagent kit (Abott, IL USA) on an Abott Architect Plus automatic biochemical analyzer (Abott, IL USA) according to the manufacturer’s manual. All the detected values were within the range of quality control requirement measured with the standards in the kits.

### Statistical analysis

Quantitative data with normal distribution was presented as mean ± standard deviation. Categorical data was presented as rate (percentage). Differences among groups were analyzed using Chi-square test or Fisher exact probability method. Receiver operating characteristic (ROC) curve analysis was used to measure the diagnostic accuracy of PIVKA- II and AFP for HBV-related HCC. Cut-off values and area under curve (AUC) were calculated. ROC curve analysis was performed with MedCalc. All other statistical analyses were accomplished using SPSS13.0 (IBM, Chicago, US). Statistical analysis was tested on two-sided settings, with *P* < 0.05 considered as statistically significant.

## Results

### HCC patients were detected with elevated PIVKA-II and AFP levels

**Fig. 1:**
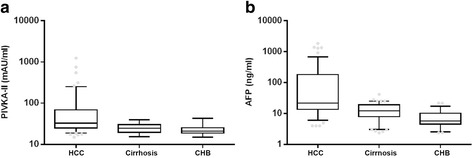
HCC patients were detected with elevated PIVKA-II and AFP levels. **a** The mean level of PIVKA-II level in HCC patients, CHB related cirrhosis patients and non-cirrhosis CHB patients was 79.64 ± 149.88, 25.29 ± 6.31 and 23.48 ± 6.71, respectively (*P* < 0.001). **b** A similar trend was found in serum AFP level among the three groups. The mean level of AFP level in HCC patients, CHB related cirrhosis patients and non-cirrhosis CHB patients was 148.62 ± 303.99, 15.16 ± 7.06 and 8.27 ± 6.03, respectively (*P* < 0.001)

Serum levels of PIVKA- II and AFP of HCC patients were compared with those of CHB patients, as shown in Fig. 1. The mean level of PIVKA-II among HCC patients was 79.64 ± 149.88, significantly higher than that of CHB-related cirrhosis patients (25.29 ± 6.31) and non-cirrhosis CHB patients (23.48 ± 6.71, *P* < 0.001). A similar trend was found in serum AFP level among the three groups. The serum levels of AFP in HCC patients, CHB-related cirrhosis patients and non-cirrhosis patients were 148.62 ± 303.99, 15.16 ± 7.06 and 8.27 ± 6.03, respectively (*P* < 0.001).

### Diagnostic value of PIVKA-II in AFP-negative HCC patients

**Fig. 2:**
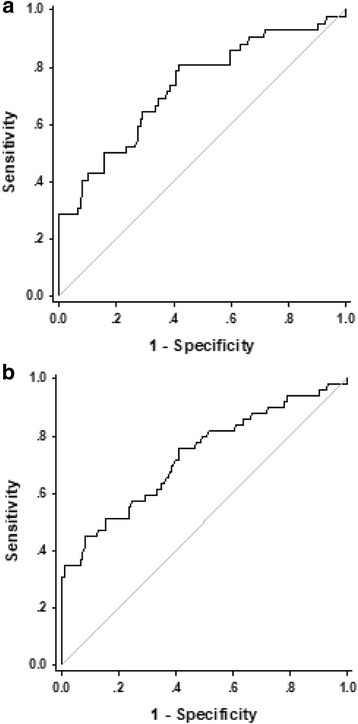
Diagnostic values of PIVKA-II in AFP-negative HCC patients and patients with small HCC. **a** The AUROC of PIVKA- II to diagnose HCC patients with small HCC was 0.692 (95%CI 0.597–0.788, *P* < 0.001). The cut-off value was 32.10 mAU/ml, the sensitivity was 54.61%, specificity was 81.92%, +LR was 3.22 and -LR was 0.59. **b** The AUROC of PIVKA-II to diagnose AFP-negative HCC patients was 0.73 (95%CI 0.640–0.815, *P* < 0.001). The cut-off value was 32.09 mAU/ml, the sensitivity was 51.02%, the specificity was 84.47%, +LR was 3.29 and -LR was 0.5

To assess the diagnostic accuracy of PIVKA-II as marker for HCC, A ROC curve was conducted. As shown in Fig. 2, patients in CHB group were pooled together as non-HCC controls. The results showed that for those AFP-negative HCC patients, the area under ROC curve (AUROC) for PIVKA-II was 0.73 (95%CI 0.640–0.815, *P* < 0.001, Fig. 2b). When the cut-off value of serum level of PIVKA-II was set as 32.09 mAU/ml, the sensitivity was 51.02%, the specificity was 84.47%, the positive likelihood ratio (+LR) was 3.29 and the negative likelihood ratio (−LR) was 0.58.

### Diagnostic value of PIVKA-II in patients with small HCC

Among HCC patients diagnosed with small HCC (tumor size ≤2 cm), the AUROC of PIVKA- II was 0.692 (95%CI 0.597–0.788, *P* < 0.001, Fig. 2a). When the cut-off value of PIVKA-II was set as 32.10 mAU/ml, the sensitivity was 54.61%, the specificity was 81.92%, the +LR was 3.22 and the -LR was 0.59. Serum level of PIVKA- II could be used for effective diagnosis of AFP-negative HCC patients and patients with small HCC.

### Diagnostic values of PIVKA-II and AFP level in HCC patient

**Fig. 3:**
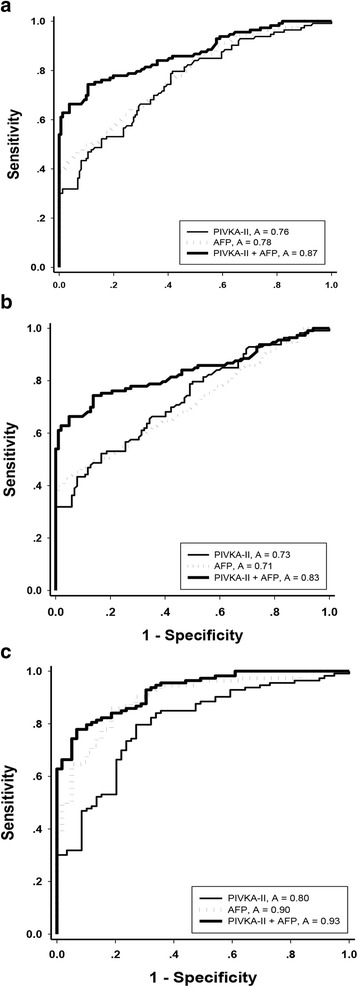
Diagnostic values of PIVKA-II and AFP in HCC patients. **a** The AUROC of PIVKA-II, AFP and PIVKA-II + AFP for diagnosis of HCC were 0.756, 0.781 and 0.868, respectively. **b** The AUROC of PIVKA-II, AFP and PIVKA-II + AFP to distinguish HCC from CHB related cirrhosis patients were 0.73, 0.71 and 0.83, respectively. **c** The AUROC of PIVKA-II, AFP and PIVKA-II + AFP to distinguish HCC from CHB patients were 0.80, 0.90 and 0.93, respectively

To evaluate the diagnostic value of PIVKA-II and AFP in HCC patients, all patients in CHB group were pooled together as control for analysis. ROC curves were plotted to identify cutoff values that would best distinguish HCC patients from controls. The AUROC of PIVKA-II was 0.756 (95%CI 0.698–0.814,*P* < 0.001), and AUROC of AFP was 0.781 (95%CI 0.726–0.836, *P* < 0.001). The optimal cutoff values of PIVKA-II and AFP were 32.09 mAU/ml and 17.56 ng/ml, respectively. When PIVKA-II was used as diagnostic marker, the sensitivity was 52.21%, specificity was 81.49%, +LR was 3.36 and -LR was 0.57. When AFP level was used as diagnostic marker, the sensitivity, specificity, +LR and –LR was 64.6%, 73.3%, 2.42 and 0.48, respectively. Serum levels of PIVKA-II and AFP were then combined to obtain a new marker for HCC diagnosis. ROC analysis showed that PIVKA-II + AFP further increased diagnostic efficiency. The AUC was 0.868 (95%CI 0.822–0.913), significantly higher than that of AFP (*P* < 0.01) or PIVKA-II (*P* < 0.001) alone. When the combined cut-off value was set as 50.23, the sensitivity and specificity was 74.34% and 89.44%, +LR and -LR was 7.04 and 0.29, respectively (Fig. 3a).

To further evaluate the diagnostic value of PIVKA-II and AFP level in distinguishing HCC patients from CHB related cirrhosis patients, CHB related cirrhosis cases were set as non-HCC controls. The AUROC of PIVKA-II, AFP and combination value was 0.73, 0.71 and 0.83, respectively. When the cut-off was set at 34.69 mAU/ml for PIVKA-II, 21.55 ng/ml for AFP and 50.23 for combination value, the sensitivities, specificities and +LR of PIVKA-II + AFP were higher than those of PIVKA-II or AFP alone. The –LR value was lower when PIVKA-II + AFP was used as diagnostic marker (Fig. 3b).

Similarly, the ROC showed that PIVKA-II, AFP and combination value could effectively distinguish HCC patients from non-cirrhosis CHB patients, as shown in Fig. 3c. The AUROC of PIVKA-II, AFP and PIVKA-II + AFP was 0.80, 0.90 and 0.93, respectively. When the cut-off value was set at 24.65 mAU/ml for PIVKA-II, 11.51 ng/ml for AFP and 44.99 for PIVKA-II + AFP, the combined PIVKA-II + AFP marker showed better diagnostic values (Fig. 3c).

### Characteristics of HCC patients in different subgroups of PIVKA-II values

According to cut-off value of 32 mAU/ml, HCC patients were divided into high PIVKA-II group and low PIVKA-II group to explorer the relationship between PIKVA-II and pathological characteristics of HCC (Table 2). Serum PIVKA-II level was higher in patients with tumors ≥2 cm than patients with tumors <2 cm. However, the difference was not significant (*p* = 0.054). PIVKA-II level was higher in poorly differentiated-undifferentiated group of patients than that in well-moderate group (*p* = 0.033). Patients in microvascular invasion group also showed higher level of PIVKA-II than patients without microvascular invasion (*p* = 0.007).

**Table 2:** Characteristics of HCC patients in different subgroups of PIVKA-II values

Characteristic	PIVKA-II > 32 mAU/ml	PIVKA-II ≤ 32 mAU/ml	*p* value
Sample size	59	54	
Gender, M/F	54/5	50/4	0.834
Age, year	47.76 ± 12.35	47.70 ± 11.12	0.979
Lab			
ALT	73.98 ± 40.89	74.06 ± 36.82	0.991
AST	76.95 ± 47.77	81.17 ± 45.61	0.633
ALB	39.48 ± 11.42	36.47 ± 10.34	0.145
TBIL	26.68 ± 12.77	27.63 ± 10.37	0.667
PLT	160.17 ± 73.63	149.22 ± 65.26	0.406
Pathology			
Cirrhosis			0.750
Yes	41	39	
No	18	15	
Tumor size			0.054
< 2	15	23	
≥ 2	44	31	
Tumor multiplicity			0.717
Single	21	21	
Multiple	38	33	
Differentiation			0.033
Well-Moderate	21	30	
Poor-Undifferentiated	38	24	
Microvascular invasion			0.007
Yes	14	3	
No	45	51	

To further confirm whether PIVKA-II is a risk factor associated with microvascular invasion in HCC patients, univariate analysis and multivariate analysis were conducted (Table 3). High serum PIVKA-II level (OR = 1.003, 95%CI 1.001–1.007, *P* = 0.047) and poorly differentiated-undifferentiated tumor (OR = 3.977, 95%CI 1.052–15.039, *P* = 0.042) were independent risk factors for microvascular invasion in HCC patients.

**Table 3:** Univariate analysis and multivariate analysis for microvascular invasion

Variables	Univariate analysis			Multivariate analysis		
	OR	95% CI	*P*	OR	95% CI	*P*
gender	0.604	0.072–5.103	0.643			
age	1.004	0.960–1.049	0.866			
PLT	0.998	0.991–1.006	0.619			
ALT	0.995	0.982–1.008	0.447			
AST	1.006	0.995–1.017	0.266			
ALB	1.029	0.987–1.073	0.177			
TBIL	1.001	0.958–1.047	0.950			
Cirrhosis	0.531	0.183–1.540	0.244			
Tumor size	0.917	0.311–2.703	0.875			
Tumor multiplicity	0.820	0.286–2.346	0.711			
Differentiation	4.667	1.260–17.288	0.021	3.977	1.052–15.039	0.042
PIVKA-II	1.004	1.001–1.008	0.034	1.003	1.001–1.007	0.047
AFP	1.001	0.998–1.002	0.668			
PIVKA-II + AFP	1.000	0.998–1.002	0.989			

## Discussion

Results of the current study showed that HBV related HCC patients contained significantly higher serum levels of PIVKA-II compared to non-HCC CHB patients. ROC analysis showed that serum PIVKA-II level was a good marker with great sensitivity and specificity to distinguish HBV related early stage HCC patients from non-HCC subjects. When combined with AFP level, PIVKA-II showed increased accuracy for diagnosing HBV related early stage HCC. Therefore, the serum level of PIVKA-II is a promising biomarker for early diagnosis of HBV related HCC.

Glutamic acid carboxylation of prothrombin precursor in the liver generates normal prothrombin which contains 10 gamma-carboxyl glutamic acid residues. This process is dependent on the presence of vitamin K. In pathological status when vitamin K is in shortage or in the presence of vitamin K dependent antagonist of carboxylase, insufficient carboxylation of glutamic acid results in production of PIVKA-II. Although the mechanism of PIVKA-II production in HCC patients is not completely clarified, it has been shown that PIVKA-II is a marker of HCC [11–13, 18, 19]. In the current study, the results showed that HBV related early stage HCC patients had higher serum levels of both PIVKA-II and AFP. The results were consistent with previous studies [12, 15, 18–20] in HCC patients, indicating that PIVKA-II served as a specific biomarker for HBV related HCC patient. Chronic hepatitis B is a high-risk factor for the development of HCC [21–23]. HBV infection could cause hepatitis flare, and eventually induce liver fibrosis, cirrhosis, and HCC, especially in those CHB patients with high HBV DNA load [24, 25]. Studies suggested that HBV infection may influence liver metabolic functions [26]. Commonly existed hyperbilirubinemia may cause malfunction of vitamin K absorbance, thus inducing abnormal levels of PIVKA-II in HBV infected subjects. Therefore, PIVKA-II is considered to be a specific biomarker for HCC instead of HBV infection.

The ROC curve analysis showed that 32mAU/ml was the optimal cutoff value of PIVKA-II for diagnosis of HBV related HCC. The optimal cutoff value of serum PIVKA-II for HCC diagnosis has been estimated to range from 30 to 42 mAU/mL in a couple of previous studies [13, 15]. Other studies have usually taken 40 mAU/mL as cutoff value when assessing the diagnostic performance of PIVKA-II as HCC biomarker [27–29]. A report used 70 mAU/ml as cutoff value to differentiate HCC patients according to the normal range and standard deviation of PIVKA-II levels in the control group [2]. In addition, it has been suggested that PIVKA-II level may vary due to pre-existing liver diseases [29]. The cutoff value of PIVKA-II for the diagnosis of HBV related HCC in the current study is slightly lower than previous reports. The reason why our cutoff value of PIVKA-II for the diagnosis of HCC was lower than previous studies might be caused by usage of different reagent systems. Besides, all the HCC patients included in this study were at early stage (BCLC 0-A). Since serum PIVKA-II levels in HCC patients will gradually increase with the progression of the disease, the inclusion of early-stage patients may also account for the difference of the optimal diagnostic cutoff value in this study and previous reports.

Previous studies on diagnostic performance of PIVKA-II for HCC have shown sensitivity ranging from 51.0% to 77%, specificity from 67.8% to 91.2%, and AUC from 0.701 to 0.854 [2, 13, 27, 28]. The diagnostic efficiency of PIVKA-II for HBV related early stage HCC is at the higher end compared to HCC diagnosis. Serum PIVKA-II and AFP are produced through different mechanisms. Therefore, the two markers are independent from each other in the diagnosis of HCC. Current results showed that PIVKA-II seemed to be a better diagnostic marker compared to AFP. We further evaluated the diagnostic performance of the two markers combined. The results showed that combination of PIVKA-II and AFP further increase the efficiency for diagnosis of HBV related early stage HCC. This result was also in consistent with a few other studies using combination of the two markers as diagnostic marker of HCC [14].

An interesting result in our study indicated that the PIVKA-II level was an independent predictor of microvascular invasion. The reason why high serum level of PIVKA-II is associated with microvascular invasion has not yet been clarified. Previous studies indicated that PIVKA-II could promote proliferation and migration of human umbilical vein endothelial cells [30]. PIVKA-II could also promote production of vascular endothelial growth factor in hepatoma cell lines [31]. Production of PIVKA-II by HCC cells promoted angiogenesis, thus inducing microvascular invasion [32]. Our results indicated that serum PIVKA-II level could be used as a biomarker for microvascular invasion, which may improve assessment of tumor prognosis and thus guide therapeutic strategy.

The current study has some limitations. Firstly, the sample size is relatively small for a practical diagnosis study. Also, there is no further cohorts to confirm the results. Secondly, the study did not differentiate cirrhosis and non-cirrhosis HCC patients, which may be a confounding factor. Since the percentages of patients with cirrhosis was not significantly different in HCC group and CHB group, we speculate that cirrhosis may not cause significant bias. Further follow-up of the patients and record of dynamic changes of PIVKA-II during treatment process may provide more information on prognostic value of biomarkers. We hope to incorporate these results in our future studies.

## Conclusion

This study demonstrates that serum level of PIVKA-II is significantly higher in HBV related early stage HCC patients compared to other HBV infected non-HCC subjects. As a biomarker, PIVKA-II has superior sensitivity and specificity for diagnosis of HBV related HCC. Combination of PIVKA-II with AFP further increases the diagnostic performance. Therefore, serum level of PIVKA-II may be considered as a screening marker for diagnosis of HBV related HCC in clinical practice.
